# Robust pyroptosis risk score guides the treatment options and predicts the prognosis of bladder carcinoma

**DOI:** 10.3389/fimmu.2022.965469

**Published:** 2022-08-24

**Authors:** Dingshan Deng, Fenglian Liu, Zhi Liu, Zuowei Wu, Yunbo He, ChunYu Zhang, Xiongbin Zu, Zhenyu Ou, Yongjie Wang

**Affiliations:** ^1^ Departments of Urology, Xiangya Hospital, Central South University, Changsha, China; ^2^ National Clinical Research Center for Geriatric Disorders, Xiangya Hospital, Changsha, China; ^3^ Departments of Laboratory Medicine, The Second Affiliated Hospital, Guizhou Medical University, Kaili, China; ^4^ Department of Interventional Radiology, Third Xiangya Hospital, Central South University, Changsha, China; ^5^ Department of Burns and Plastic Surgery, Changsha, China

**Keywords:** bladder cancer, pyroptosis, risk score, tumor immune microenvironment, immunotherapy, chemotherapy, radiotherapy

## Abstract

**Background:**

Bladder carcinoma (BLCA) is a heterogeneous disease that makes it difficult to achieve proper individual treatment and predict prognosis. This study aimed to develop a risk score from a new perspective of pyroptosis and guide accurate treatment and prognosis prediction for BLCA.

**Methods:**

The TCGA-BLCA cohort data were downloaded from The Cancer Genome Atlas database. Two external validation cohorts were collected from the Gene Expression Omnibus. Another independent validation cohort (the Xiangya cohort) was recruited from our hospital. The least absolute shrinkage and selector operation (LASSO) algorithm and Cox regression models were used to establish the pyroptosis risk score. Thereafter, we correlated the pyroptosis risk score with prognosis, tumor microenvironment (TME) immune hallmarks, and multiple treatments, including anticancer immunotherapy, chemotherapy, radiotherapy, and targeted therapy.

**Results:**

The pyroptosis risk score was an independent prognostic predictor of BLCA. We found that the activities of multiple steps of the anticancer immune response cycle, such as the release of cancer cell antigens, CD8 T cell recruitment, and NK cell recruitment, were significantly higher in the high-risk score group than in the low-risk score group. In addition, the infiltration levels of the corresponding tumor-infiltrating immune cells (TIICs), such as CD8 T cells and NK cells, were positively correlated with the pyroptosis risk score. Thus, BLCA with a high-risk score may be associated with inflamed phenotypes. Simultaneously, the expression of multiple immune checkpoints (such as PD-L1, CTLA-4, and PD-1) and enrichment scores of gene signatures positively correlated with immunotherapy response were positively correlated with the pyroptosis risk score. Therefore, patients with a high pyroptosis risk score may be more sensitive to immunotherapy. In addition, patients with high pyroptosis risk scores may be more sensitive to chemotherapeutic drugs, such as cisplatin, docetaxel, and paclitaxel. In addition, the pyroptosis risk score accurately predicted the molecular subtypes of BLCA, which were cross-validated in several independent systems.

**Conclusions:**

This study developed and validated a robust pyroptosis risk score that can predict the clinical outcomes and TME immune phenotypes of BLCA. In summary, the pyroptosis risk score helps drive precision therapy in patients with BLCA.

## Introduction

Bladder cancer is one of the most common urinary tumors with an increasing incidence. Approximately 150,000 people worldwide die of this disease every year (1). Non-muscle invasive bladder cancer (NMIBC) can be treated with surgical resection and intravesical perfusion therapy; however, most patients still experience recurrence (2). In muscle invasive bladder cancer (MIBC), the main treatments include surgery, radiotherapy, targeted therapy, and anticancer immunotherapy (2, 3). However, these treatment options are insufficient to cure BLCA. Only a minority of patients are sensitive to these regimens, which are caused by many primary or acquired resistance mechanisms such as pyroptosis (4). The inherent genetic heterogeneity of tumor cells and metabolism-related factors cause tumor cells to acquire drug resistance to treatments (4). Bladder cancer is a heterogeneous tumor with many molecular subtypes, making it difficult to achieve accurate treatment (2). Therefore, it is important to develop effective tools to reveal the heterogeneity of BLCA and predict its prognosis and efficacy.

Pyroptosis is a programmed inflammatory cell death usually accompanied by the activation of inflammatory bodies and maturation of proinflammatory cytokines IL-1 β (IL-1 β) and interleukin-18 (IL-18) (4, 5). In recent years, researchers have conducted several studies on tumor cell pyroptosis. Pyroptosis inhibits tumor growth in colorectal, liver, skin, and other cancers (6). The role of pyroptosis in bladder tumors requires further investigation. Some studies have shown that GSDME, a member of the gasdermin superfamily, can trigger pyroptosis by cleaving GSDMD by activating caspase-3 during chemotherapy (7). Caspase 8 is considered a molecular switch that regulates pyroptosis (8). Pyroptosis affects tumor proliferation, invasion, and metastasis, reshapes the tumor microenvironment, and stimulates anti-tumor immune responses. Some molecules related to pyroptosis have been identified in some tumors and can be used to predict prognosis and therapeutic response (5, 9). Current immunotherapy, including anti-CTLA-4, anti-PD-1/PD-L1, and chimeric antigen receptor (CAR) T-cell therapy, has significantly improved the survival outcomes of patients with cancers (10–12). However, the relationship between pyroptosis and the tumor immune microenvironment in BLCA needs to be further explored.

This study integrated several independent BLCA datasets and developed a novel pyrolysis risk score. We correlated the pyrolysis risk score with clinical prognosis, the tumor microenvironment (TME) phenotypes, and response to multiple treatment regimens.

## Materials and methods

### Data sets collection

#### External public cohorts

The mRNA expression matrix (FPKM) of 414 BLCA tumor samples and 19 normal tissues were downloaded from the TCGA Cancer Genome Atlas (https://portal.gdc.cancer.gov/). Thereafter, the FPKM values were converted to TPM values. Two externally validated GSE cohorts with detailed survival data, GSE32894 and GSE48075 were collected from the Gene Expression Omnibus (GEO). GSE32894 (platform: GPL6947) contained 224 BLCA samples and GSE48075 (platform: GPL6947) contained 73 BLCA samples with survival information.

Xiangya cohort: According to our previous study, the Xiangya cohort (GSE188715) comprised 57 BLCA samples sequenced on the BGISEQ-500 platform (BGI-Shenzhen, China) (13–15). Determination of pyroptosis gene sets: The keyword “pyroptosis” was searched in the GSEA public database (http://www.gsea-msigdb.org/gsea/msigdb/genesets.jsp). Two gene sets, “GO BP_PYROPTOSIS” and “REACTOME_PYROPTOSIS, “ were obtained. Forty pyroptosis-related genes were also identified. In addition, we extracted 33 pyroptosis-related genes from previous studies (16–19). Finally, 57 pyroptosis-related genes were obtained (**Supplementary Table 1**).

The detailed information of these cohorts was provided in **Supplementary Table 1**.

### Single cell RNA sequencing (scRNA-seq)

Three muscle-invasive bladder cancer (MIBCs) samples were obtained from the Department of Urology, Xiangya Hospital, and scRNA-seq was performed at OE Biotech Co, Ltd (Shanghai, China), named as the Xiangya scRNA cohort. There are studies reporting that the detailed preparation of single-cell suspensions is based on droplet processing of raw data and single-cell sequencing (20, 21). After processing through cell ranger, the Seurat R package (version 4.1.0) was used to convert the count matrix to a Seurat object. Low-quality cells were cells with a unique molecular identifier (UMI) number of less than 1000, a gene number of less than 200, a log10GenesPerUMI number of less than 0.70, and a mitochondrial-derived UMI number of more than 20%, and these cells were discarded. The count matrixs were then normalized and the effects of mitochondrial proportion were regressed. The functions SelectIntegrationFeatures, findinintegrationanchors and IntegrateData integrate these three samples based on the first 3000 variable features. Afterwards, principal component analysis (PCA) was used to display the cell clusters through the tSNE plot and use the FindClusters function to screen the main cell clusters (res=0.4). To identify malignant bladder cancer cells, CNVs in epithelial cells were screened by the InferCNV package.

### Identification of differentially expressed pyroptotic genes (pyroptotic DEGs) and functional analysis

The empirical Bayesian method of the limma R package was used to identify differentially expressed pyroptotic DEGs between bladder cancer and normal tissues. The screening criteria for pyroptotic DEGs were as follows: |log(fold change)|>1 and adjusted P-value< 0.05 (22). The Kyoto Encyclopedia of Genes and Genomes (KEGG) and Gene Ontology (GO) analyses were performed using the aforementioned pyroptotic DEGs (23).

### Development and validation of pyroptosis risk score

First, pyroptotic DEGs from cancerous and paracancerous tissues were screened in the TCGA-BLCA cohort. Thereafter, we screened the prognostic pyroptotic genes in the TCGA-BLCA cohort using univariate Cox analysis. Furthermore, least absolute shrinkage and selection operator (LASSO) regression was performed to identify the pyroptotic DEGs with the best prognosis. Finally, based on the best prognostic pyroptotic DEGs, the risk score for pyroptosis was calculated using the LASSO coefficient: Risk score = ∑ βi _*_ RNAi, where βi is the coefficient of the i-th.

Patients were divided into high- and low-scoring groups according to the median pyroptosis risk score. Kaplan-Meier survival analysis was used to obtain the survival curves. The predictive prognostic accuracy of pyroptosis risk score was determined using the tROC R package. The prognostic accuracy of the pyroptosis risk score was validated in the GSE32894, GSE48075, and an internal cohort (Xiangya cohorts). In addition, the pyroptosis risk score was correlated with the grade and stage of tumors. Univariate and multivariate Cox analyses were used to analyze the independent prognostic role of sex, age, stage, and the pyroptosis risk score in the TCGA-BLCA cohort. Finally, a nomogram was plotted based on these factors with an independent prognostic predictive value. The nomogram was validated using clinical decision curves.

### Description of BLCA molecular subtypes and TME characteristics

In our previous study, seven independent molecular subtype systems were analyzed, including the UNC, Baylor, TCGA, MDA, CIT, and consensus systems (15). Relevant molecular subtype-specific signatures were collected and correlated with pyroptosis risk scores. In our previous study, the related immunological characteristics and algorithms in the TME were described in detail (13–15, 20, 24). The steps of the cancer-immune cycle include cancer antigen presentation, release, immune cell trafficking, recognition, and killing. Thereafter, various independent algorithms, such as TIP, CiberSort-ABS, and TIMER, were used to obtain the infiltration degree of tumor-infiltrating immune cells (TIIC) (13–15, 20, 24).

### Gene set variation analysis (GSVA) and response prediction of several treatment options

GSVA is often used to estimate the activity differences of pathways or biological processes in expression dataset samples and is a nonparametric unsupervised method (25). To study the differences of 50 correlation pathways among the pyroptosis risk score groups, the corresponding paths from MSigDB and analyzed GSVA enrichment were collected using “GSVA” R software package (26). Individualized chemotherapy response was estimated using the pRRophetic software package based on data from the Genomics of Cancer Drug Sensitivity (GDSC) (https://www.ancerrxgene.org/) (27). We calculated the IC50 values of cisplatin, docetaxel, paclitaxel, bleomycin, camptothecin, and vinblastine. In addition, we summarized some effective indicators for predicting the efficacy of ICB, including the pan-cancer T cell inflammation score (TIS) and 20 inhibitory immune checkpoints (15). Finally, the enrichment fraction of the signature related to clinical response to targeted therapy and radiotherapy was calculated using the ssGSEA algorithm.

### Statistical analysis

Statistical analysis of all relevant data was performed using the R software. Pearson or Spearman coefficients were used to analyze the correlations between variables. The t-test or Mann-Whitney U test was used to compare differences in continuous variables between the groups. Survival curves were plotted using the Kaplan-Meier method. We used receiver operating characteristic (ROC) curves to calculate the accuracy of pyroptosis risk scores for predicting survival and molecular subtypes. Statistical tests were two-sided, and the level of significance was set at P< 0.05.

## Results

### Identifying differentially expressed genes between bladder cancer and normal tissues

**Figure 1:**
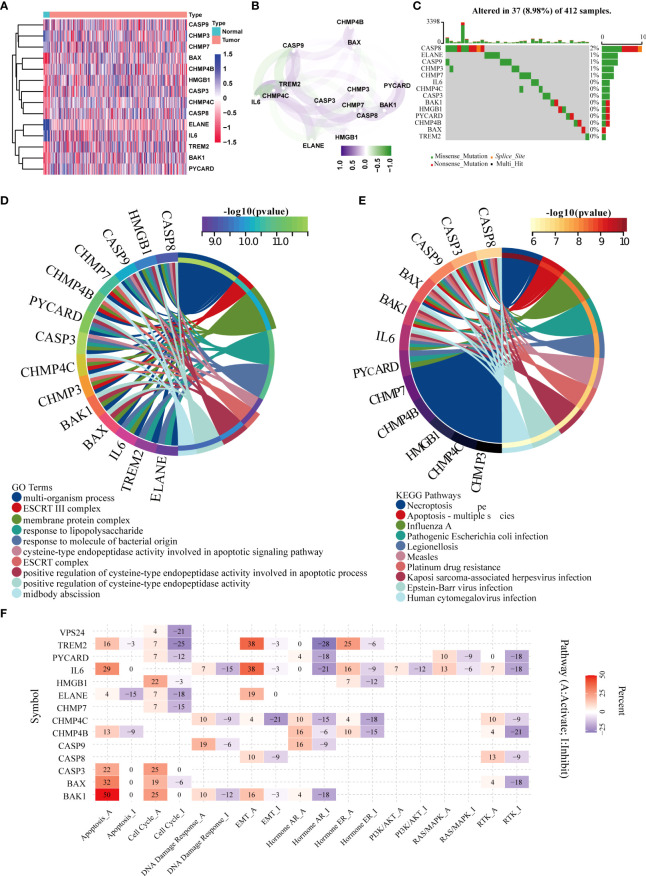
Screening of differentially expressed pyroptotic genes and functional analysis. **(A)** Fourteen pyroptotic genes differentially expressed in BLCA and normal tissues. **(B)** protein–protein interaction (PPI) network of the differentially expressed pyroptotic genes. **(C)** Landscape of mutation profiles in 412 patients with bladder cancer from the TCGA-BLCA cohort. Each waterfall plot represents the mutation information of each pyroptosis-related regulator. Corresponding colors have annotations at the bottom, indicating different mutation types. The barplot shows mutation burden. The right numbers represent mutation frequency individually. **(D)** GO analysis of the differentially expressed pyroptotic genes. The corresponding colors have annotations at the bottom, indicating different biological pathways. **(E)** KEGG analysis of the differentially expressed pyroptotic genes. The corresponding colors have annotations at the bottom, indicating different pathways. **(F)** Heatmap showing correlations between 14 pyroptotic genes and their expression levels in important cancer signaling pathways. Red represents the activated pathway, whereas blue represents the inhibitory pathway.

Among the 57 pyroptosis-related genes, 14 were differentially expressed between BLCA and adjacent normal tissues (**Figure 1A**), including CASP9, CHMP3, CHMP7, BAX, CHMP4B, HMGB1, CASP3, CHMP4C, CASP8, ELANE, IL6, TREM2, BAK1, and PYCARD. Meanwhile, we further analyzed the expression patterns of these DEGs in the BLCA microenvironment from the single cell level. First, it was found that seven genes, BAK1, CASP3, CASP8, CASP9, CHMP7, ELANE, and IL6, were not specifically expressed in each cell line (**Supplementary Figure 1**). In contrast, the genes HMGB1, PYCARD and BAX were expressed in all cell lines without specificity (**Supplementary Figure 4**). Second, two genes, CHMP4B and CHMP3, were not expressed in immune cells (T/NK and B cells) (**Supplementary Figure 2**). Third, the CHMP4C was specifically expressed in bladder cancer epithelial cells (**Supplementary Figure 3**). Last, the gene TREM2 was found to be specifically expressed in myeloid cell lines (**Supplementary Figure 5**).

**Figure 1B** shows the correlation network diagram of the 14 pyroptotic DEGs, and the results show that most of the genes were related to each other. To observe the genetic variation in pyroptotic molecules in bladder cancer, we displayed the somatic mutation frequencies of 14 differentially expressed pyroptotic molecules in the TCGA-BLCA cohort using waterfall plots. Among the 412 BLCA samples, pyroptotic mutations were found in 37 cases with a mutation frequency of 8.98%. We found that CASP8 had the highest mutation frequency (2%), of which missense mutations were the most common. In summary, the mutation frequencies of these pyroptosis-related genes were low (**Figure 1C**). Therefore, we performed a functional enrichment analysis based on these 14 pyroptosis DEGs. GO analysis showed that these differentially expressed pyroptotic genes were enriched in multiple biological pathways, including multi-organism processes, ESCRT III complex, and membrane proteins (**Figure 1D**). KEGG analysis showed that these pyroptosis genes were enriched in Necroptosis, Apoptosis and Influenza A (**Figure 1E**). Furthermore, we performed pan-cancer analysis based on the 14 differentially expressed pyroptosis genes. The results suggested that 14 pyroptosis genes were closely related to nine important tumor-related pathways in pan-cancer (**Figure 1F**). We found that most pyroptosis genes were related to activation of the apoptosis pathway. For example, BAK1, BAX, and IL6 are associated with the activation of apoptosis in 50%, 32%, and 29% of tumors, respectively. In contrast, only ELANE was associated with apoptosis inhibition in 15% of tumors. Several other obvious pathways have been described in the manner of apoptosis: cell cycle, EMT, hormone AR, hormone ER, and RTK.

### Pan-cancer multi-omics analysis of pyroptotic DEGs

We further analyzed the multi-omics features of the 14 pyroptotic DEGs in pan-cancer. The results suggested that these genes had high mutation frequencies in UCEC, HNSC, STAD, COAD, and BLCA. Among them, the mutation frequency of the CASP8 gene was the highest at 55% in UCEC (**Supplementary Figure 6A**). In addition, we found that TREM2, CASP3, BAX, CASP8, PYCARD, CHMP4C, and BAK1 were highly expressed in most tumors compared to paracancerous tissues. Conversely, IL6 and ELANE were downregulated in most tumors compared to adjacent tissues (**Supplementary Figure 6B**). The main types of copy number variations of pyroptosis genes in pan-cancer were heterozygous amplifications and deletions, among which the CNVs of CHMP4B, CHMP4C, and IL6 in most tumors were heterozygous amplifications. In contrast, CASP3, CASP9, and CHMP7 had CNV-type loss-of-heterozygosity in most tumors (**Supplementary Figure 6C**). We found that an important factor affecting the expression of pyroptotic molecules was gene copy number variation, as CNV and mRNA expression levels were positively correlated in most tumor types (**Supplementary Figure 6E**). In particular, for CHMP7, there was a significant positive correlation between CNV and mRNA expression in most tumors (**Supplementary Figure 6E**). Furthermore, in most cancers, the methylation levels of the aforementioned pyroptotic genes were negatively correlated with the mRNA expression levels (**Supplementary Figure 6D**).

### Development and validation of a pyroptosis risk score in the TCGA-BLCA cohort

**Figure 2:**
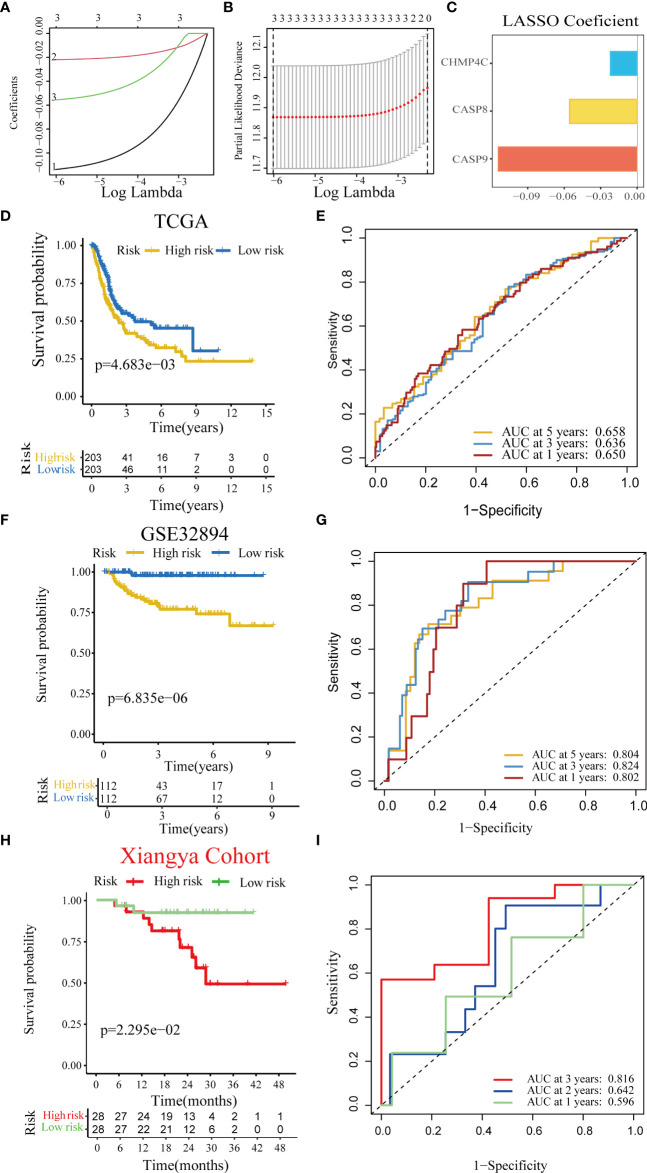
Construction and validation of a pyroptosis risk score in the multiple BLCA cohorts. **(A)** Lasso coefficients of 14 predicted pyroptotic genes in the TCGA-BLCA cohort. **(B)** Cross-validation for turning parameter selection *via* minimum criteria in the LASSO regression model. **(C)** Three best candidates were screened by LASSO algorithm to further determine the generation of the pyroptosis risk score. **(D)** Kaplan–Meier analysis of OS for the pyroptosis risk score in the TCG-BLCA cohort. **(E)** ROC curves of the pyroptosis risk score for predicting OS in the TCG-BLCA cohort. **(F, G)** Validation of the pyroptosis risk score in GSE32894. **(H, I)** Validation of the pyroptosis risk score in the Xiangya cohort.

First, we performed a univariate Cox regression analysis based on 14 pyroptotic DEGs in the TCGA-BLCA cohort. Univariate Cox regression analysis revealed that CASP9, CHMP4C, and CASP8 were associated with prognosis. Furthermore, we identified three optimal candidates for constructing a pyroptosis risk score using the LASSO algorithm (**Figures 2A−C**). In the TCGA training cohort, we divided the patients into low-and high-risk score groups based on the median risk score. The results showed that the overall survival time of patients in the low-score group was significantly longer than that in the high-score group (**Figure 2D**). The AUC of the pyroptosis risk score for predicting the OS of bladder cancer OS was 0.650, 0.636, and 0.658 at 1, 3, and 5 years, respectively (**Figure 2E**). In the independent external validation cohort GSE32894, patients in the low-score group also had a significantly longer overall survival than those in the high-score group (**Figure 2F**). The AUC of the pyroptosis risk score for predicting bladder cancer OS were 0.802, 0.824, and 0.804 at one, three, and five years, respectively (**Figure 2G**). We found consistent results in the Xiangya internal validation cohort. The patients in the low-scoring group had a better overall prognosis (**Figure 2H**). The AUC of the pyroptosis risk score for predicting the OS of bladder cancer OS was 0.596, 0.642, and 0.816 at 1, 3, and 5 years, respectively (**Figure 2I**).

### Relationship between pyroptosis risk score and clinicopathological features

**Figure 3:**
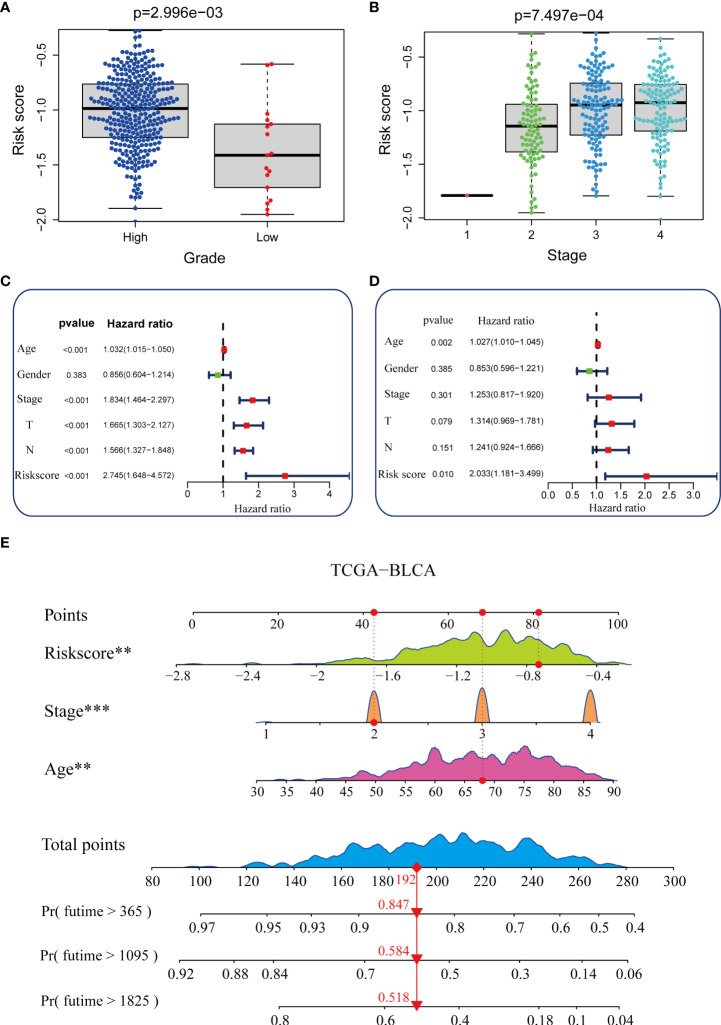
Construction of a nomogram in the TCGA-BLCA cohort**. (A, B)** Relationship between the pyroptosis risk score and tumor grade and stage in the TCGA-BLCA cohort. **(C, D)** Results of univariate and multivariate Cox analyses. **(E)** Nomogram developed based on stage, age, and the pyroptosis risk score to predict overall survival at 1, 3, and 6 years. **P value < 0.01, ***P value < 0.001.

**Figure 4:**
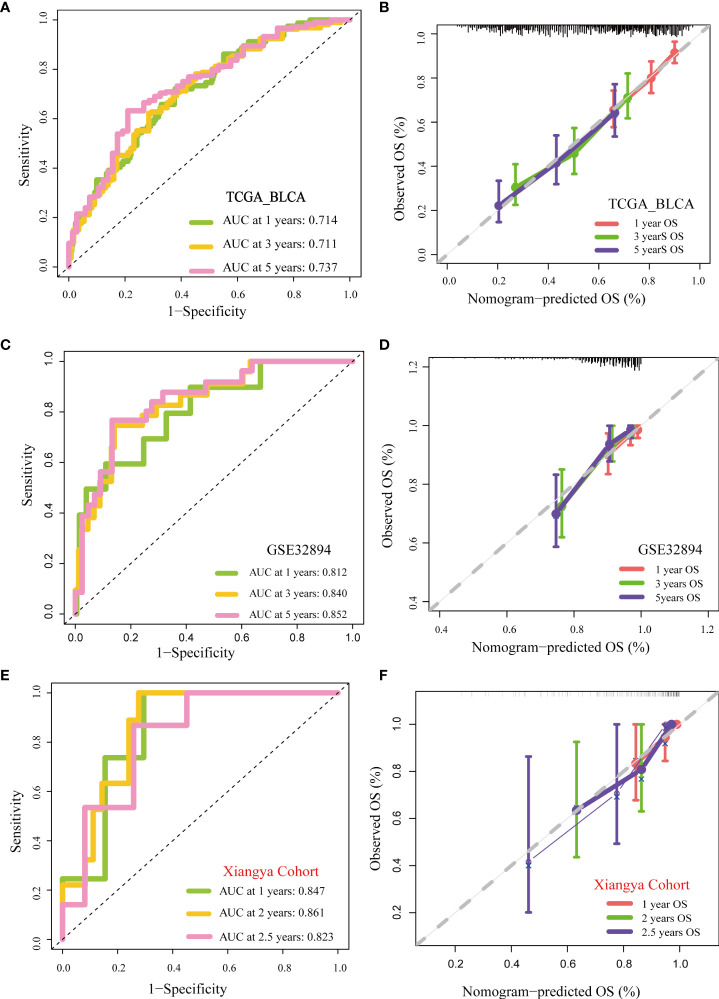
Validation of multiple cohorts of the pyroptosis risk score **(A)** ROC curves of the nomogram. **(B)** Calibration curves of the nomogram measured using the Hosmer–Lemeshow test. **(C, D)** Validity of pyroptosis risk score in GSE32894. **(E, F)** Validity of the pyroptosis risk score in the Xiangya cohort.

As shown in **Figures 3A, B**, patients with higher grades and stages had higher risk scores, which was consistent with the prognostic correlation of pyroptosis risk scores. Muscle invasive status, metastasis and histological type also had the same relationship with pyroptosis risk score **(** **Supplementary Figure 7** **)**.Furthermore, univariate Cox analysis suggested that age, stage, and the pyroptosis risk score were significant prognostic predictors (**Figure 3C**). Further multivariate Cox analysis confirmed that the pyroptosis risk score was an independent prognostic risk factor (**Figure 3D**). However, staging no longer has an independent prognostic predictive value. These results demonstrate that the pyroptosis risk score is an effective indicator for predicting the prognosis of patients with BLCA. To improve the predictive value of the pyroptosis risk score for the prognosis of bladder cancer, we established a comprehensive line chart by combining the pyroptosis risk score with several factors that had prognostic value in the univariate Cox regression analysis, such as age and tumor stage. Figure (**Figure 3E**). We further used the ROC and calibration curves to verify the accuracy of the line chart in predicting the prognosis of bladder cancer. In the TCGA-BLCA cohort, the prediction accuracies of the line charts for 1-, 3-, and 5-year OS were 0.714, 0.711, and 0.737, respectively (**Figure 4A**). As shown in the calibration curve (**Figure 4B**), the OS predicted by the line chart was highly consistent with the actual OS, highlighting the clinical significance and accuracy of this comprehensive line chart. More importantly, the line chart showed a higher prognosis prediction accuracy in the two verification sets, GSE32894 and Xiangya cohorts. (**Figures 4C−F**).

### Pyroptosis risk score were related to TME immune characteristics and ICB clinical response

**Figure 5:**
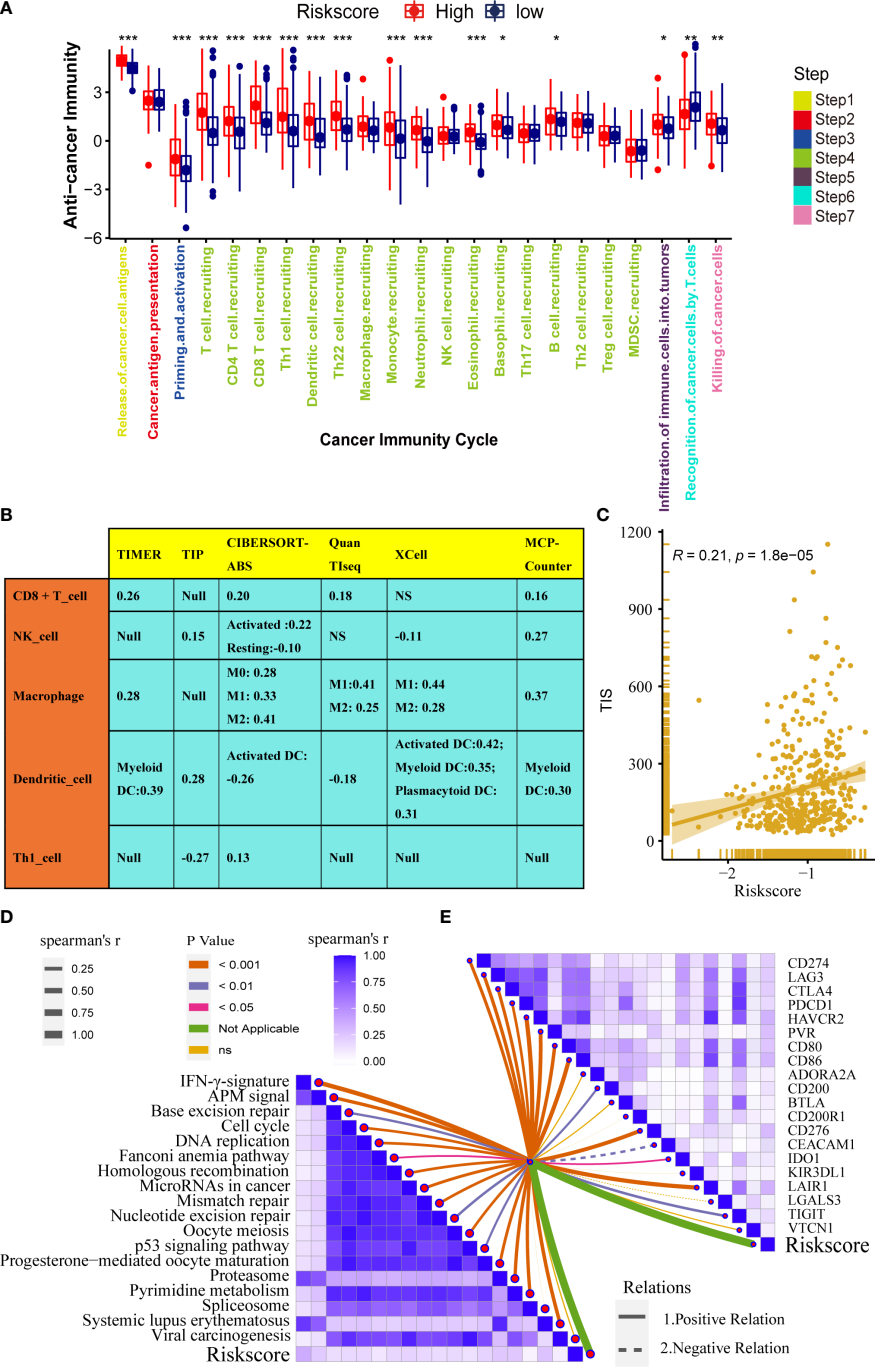
Pyroptosis risk score correlated with the tumor immune microenvironment characteristics. **(A)** Differences in cancer immune cycling activity between high- and low-risk groups. **(B)** Relationships between the pyroptosis risk score and several immune cells (CD8+T cells, NK cells, macrophages, Th1 cells, and DCs) in six independent algorithms. **(C)** Relationships between the pyroptosis risk score and T cell inflamed score (TIS). **(D)** Correlation between the pyroptosis risk score and enrichment of ICB response-related pathways. **(E)** Relationship between pyroptosis risk scores and immune checkpoints (*P < 0.05; **P < 0.01; ***P < 0.001). NS, P value > 0.05, no significant difference;.

Immunotherapy has become the first-line treatment for advanced bladder cancer. Therefore, it is crucial to explore the correlation between the pyroptosis risk score and the immune microenvironment of bladder cancer. The fate of cancer cells and the efficacy of immunotherapy depend on the state of the tumor immune microenvironment. The whole cancer immune cycle consists of a series of continuous steps (28), the seven main steps were: release of cancer cell antigens, cancer antigen presentation, priming and activation, trafficking of immune cells to tumors, infiltration of immune cells into tumors, recognition of cancer cells by T cells, and killing of cancer cells (15). We analyzed the correlation between risk score and activities of several anticancer immune steps. The results suggested that the activities of release of cancer cell antigens, T cell recruitment, CD8 T cell recruitment, Th1 cell recruitment, NK cell recruitment, and other steps were significantly higher in the high-score group than in the low-score group (**Figure 5A**). Consistently, the infiltration levels of the corresponding TIICs, such as CD8 T cells, NK cells, Th1 cells, and dendritic cells, were positively correlated with the pyroptosis risk score. These results were highly consistent across the six independent algorithms (**Figure 5B**). These results suggest that patients in the high-risk score group may have an inflammatory phenotype that is more sensitive to ICB. Therefore, we correlated the risk score with several predictors of ICB efficacy. We found a significant positive correlation between the risk scores and TIS (**Figure 5C**). In addition, the expression of many immune checkpoints (such as CD274, CTLA4, and PDCD1) and the enrichment score of immunotherapy-related gene signatures were positively correlated with the risk score (**Figures 5D, E**).

In summary, high-risk score tumors are inflamed phenotypes that are more sensitive to ICB.

### Pyroptosis risk score accurately predicts molecular subtypes and promotes precision medicine for BLCA

**Figure 6:**
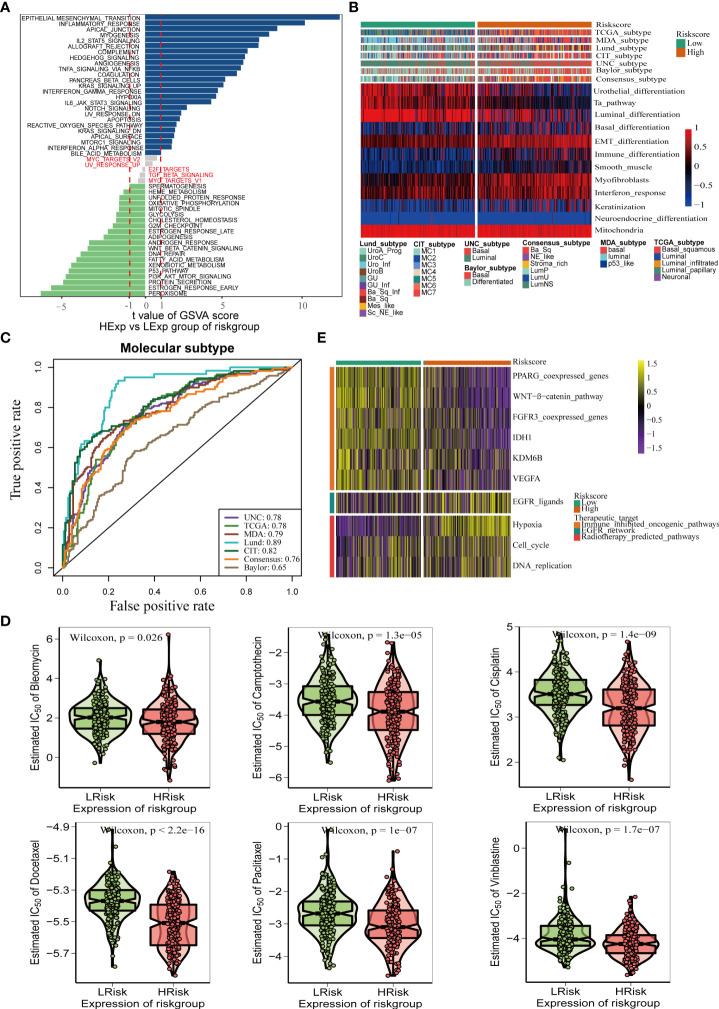
Pyroptosis risk score effectively predicts molecular subtypes and guides precise treatment of BLCA. **(A)** Differences in biological function between pyroptosis risk groups. **(B)** Relationships between pyroptosis risk score and seven classical molecular subtypes. **(C)** Predictive accuracy of pyroptosis risk score for molecular subtypes in multiple different algorithms. **(D)** Difference on the effects of six chemotherapy drugs. **(E)** Relationships between the pyroptosis risk score and enrichment scores of multiple therapeutic signatures.

Bladder cancer comprises a variety of molecular subtypes with significantly different biological functions. Therefore, we first compared the differences in the enrichment activities of the 50 hallmark signaling pathways between the high- and low-risk groups. We found significantly different biological functions between the high- and low-pyroptosis score groups (**Figure 6A**). EPITHELIAL MESENCHYMAL TRANSITION and INFLAMMATORY RESPONSE were the most abundant signals in the high-risk group, whereas PEROXISOME and ESTROGEN RESPONSE_EARLY were the most abundant signals in the low-score group. These results suggest that pyroptosis genes may affect the progression of BLCA by regulating hallmark signaling pathways. Thereafter, we analyzed the correlation between risk score and molecular typing of bladder cancer. The results showed that the high-scoring group was mostly the basal subtype characterized by basal differentiation, EMT differentiation, immune differentiation, myofibroblasts, and interferon response, whereas the low-scoring group was mainly the luminal subtype characterized by the Ta pathway and luminal differentiation (**Figure 6B**). **Figure 6C** shows that the risk score of pyroptosis could accurately predict molecular subtypes, and the AUC for predicting molecular subtypes in UNC, TCGA, MDA, Lund, CIT, consensus, and Baylor were 0.78,0.78,0.79,0.89,0.82,0.76, and 0.65, respectively. Different molecular types have different sensitivities to different treatments, including radiotherapy, chemotherapy, and targeted therapies. Therefore, we further analyzed the sensitivity of the pyroptosis risk score in predicting the most frequently used chemotherapeutic drugs among the six BLCA. Patients with high scores were more sensitive to chemotherapy drugs including cisplatin, camptothecin, paclitaxel, bleomycin, docetaxel, and vinblastine (**Figure 6D**). Finally, we found that the enrichment activity of gene signatures related to the efficacy of EGER-targeted therapy and radiotherapy was higher in the high-score group, indicating that patients in the high-score group were sensitive to EGER-targeted therapy and radiotherapy (**Figure 6E**).

### To verify the role of pyroptosis risk score in Xiangya cohort, GSE32894, and GSE48075

**Figure 7:**
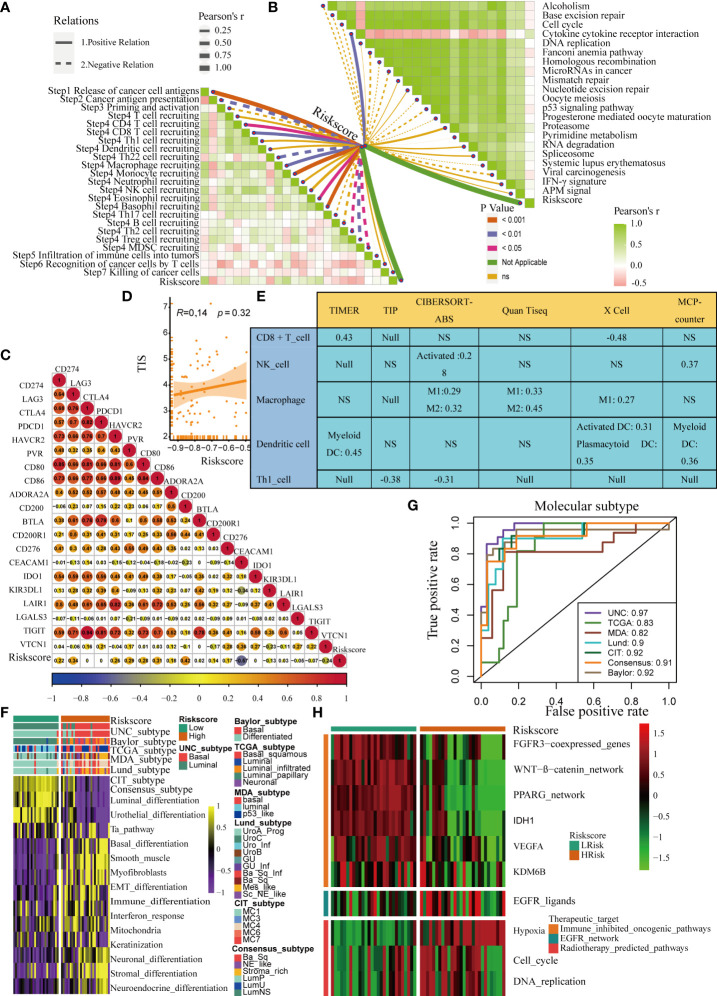
Validation of the pyroptosis risk score in the Xiangya cohort. **(A)** Relationships between the pyroptosis risk score and activities of the cancer immunity cycles. **(B)** Relationships between the pyroptosis risk score and immunotherapy-predicted pathways. **(C)** Correlations between the pyroptosis risk score and several immune checkpoints. **(D)** Relationships between the pyroptosis risk score and T cell inflammation score (TIS). **(E)** Relationship between the pyroptosis risk score and infiltration levels of five tumor-infiltrating immune cells. **(F)** The pyroptosis risk score accurately stratified the molecular subtypes in seven different algorithms. **(G)** Accuracy of the pyroptosis risk score in predicting molecular subtypes in seven different algorithms. **(H)** Relationships between the pyroptosis risk score and the enrichment scores of several therapeutic signatures. NS, P value > 0.05, no significant difference.

**Figure 8:**
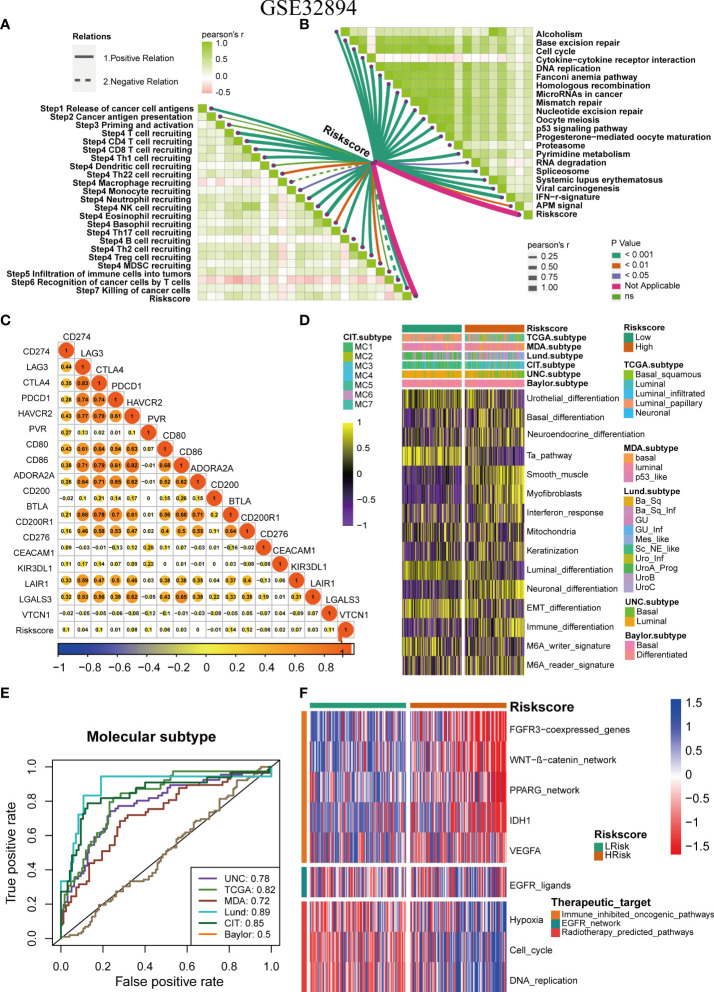
Validation of the pyroptosis risk score in the GSE32894 cohort. **(A)** Relationships between pyroptosis risk score and the activities of the cancer immunity cycles. **(B)** Relationships between the pyroptosis risk score and immunotherapy-predicted pathways. **(C)** Correlations between the pyroptosis risk score and several immune checkpoints. **(D)** The pyroptosis risk score accurately stratified the molecular subtypes in seven different algorithms. **(E)** Accuracy of the pyroptosis risk score in predicting molecular subtypes in seven different algorithms. **(F)** Relationships between the pyroptosis risk score and enrichment scores of several therapeutic signatures.

**Figure 9:**
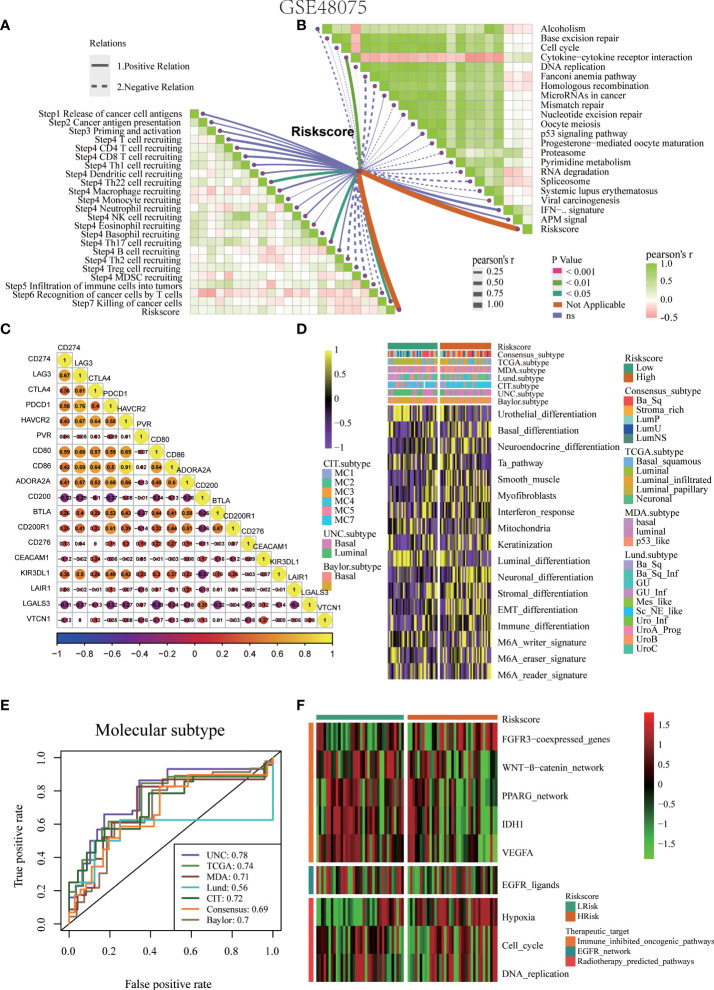
Validation of the pyroptosis risk score in the GSE48075 cohort. **(A)** Relationships between the pyroptosis risk score and activities of the cancer immunity cycles. **(B)** Relationships between the pyroptosis risk score and immunotherapy-predicted pathways. **(C)** Correlations between the pyroptosis risk score and several immune checkpoints. **(D)** The pyroptosis risk score accurately stratified the molecular subtypes in seven different algorithms. **(E)** Accuracy of the pyroptosis risk score in predicting molecular subtypes in seven different algorithms. **(F)** Relationships between the pyroptosis risk score and enrichment scores of several therapeutic signatures.

**Figure 10:**
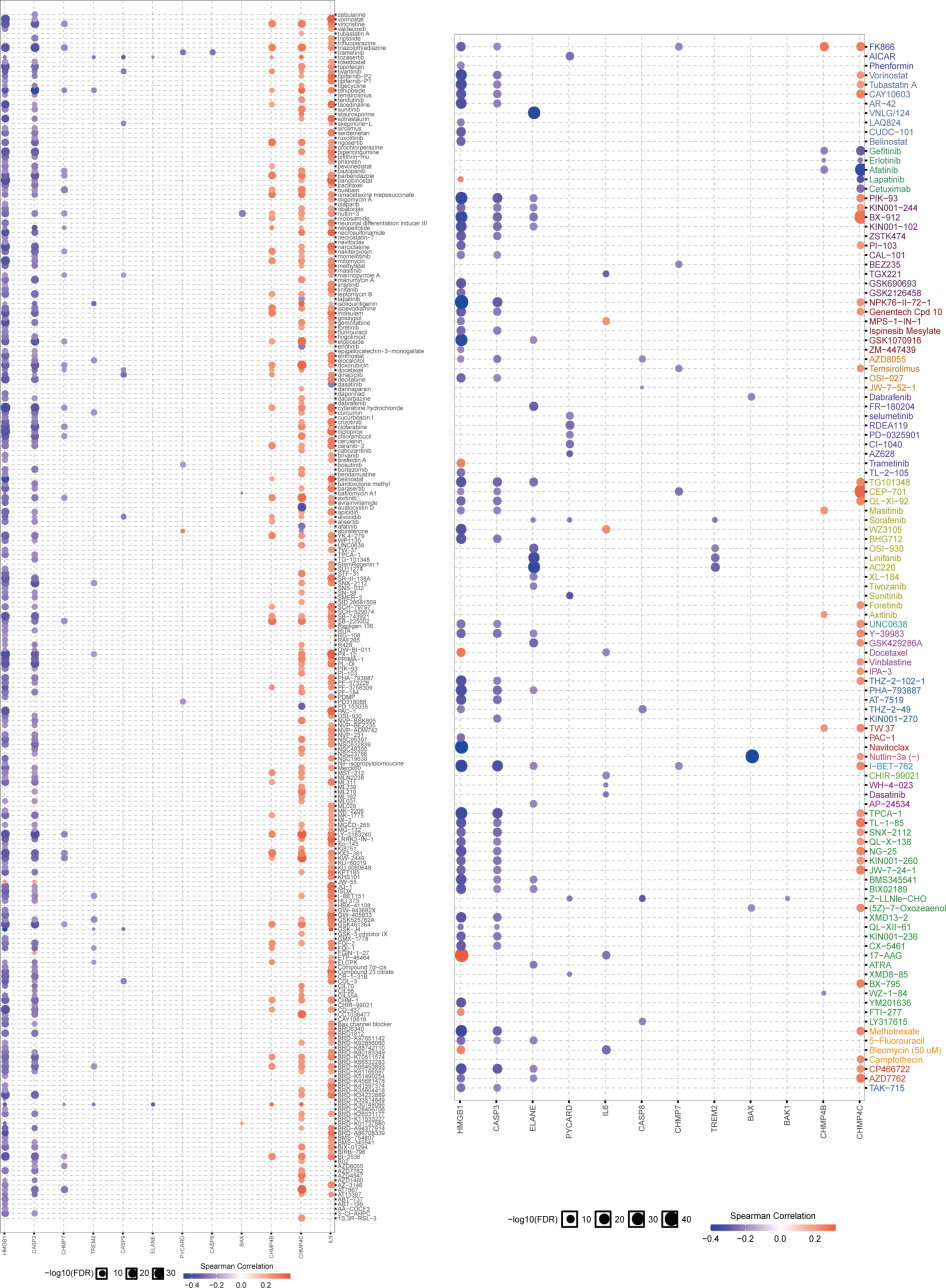
Correlation analysis between pyroptosis-related genes and drug sensitivity. **(A, B)** Bubble chart shows the correlation analysis between these pyroptotic genes and drug susceptibility. Red indicates positive correlation and blue indicates negative correlation. The darker the color, the higher the correlation index. Bubble size indicates the FDR.

The role of the pyroptosis risk score in predicting immune-related phenotypes, molecular subtypes, and treatment regimen efficacy was further validated in the Xiangya cohort. The pyroptosis risk score was positively correlated with the enrichment scores for most steps of the anticancer immune cycle (**Figure 7A**). Consistently, the pyroptosis risk score was positively correlated with the corresponding degree of TIIC infiltration by CD8 + T cells, NK cells, and dendritic cells (**Figure 7E**). Enrichment scores for signaling pathways positively correlated with immune checkpoint, TIS, and ICB responses were also positively correlated with the pyroptosis risk scores (**Figures 7B−D**). Therefore, in the Xiangya cohort, tumors with high-risk scores also belonged to the inflammatory phenotype. In addition, in the Xiangya cohort, the pyroptosis risk score was accurate for the molecular subtypes (**Figure 7F**). In these seven independent systems, the AUC ranged from 0.83 to 0.97 (**Figure 7G**). As expected, in the Xiangya cohort, the pyroptosis risk score could also accurately predict the effects of radiotherapy and several targeted therapies, and patients in the high score group were more sensitive to EGFR-targeted therapy and radiotherapy; targeted therapy such as blockade of the FGFR3 network, WNT-b-catenin network, and PPRAG network were more sensitive to low-score patients (**Figure 7H**). All the above results were effectively validated in GSE32894 and GSE48075 (**Figures 8A−F** and **9A–F**). Finally, we correlated the 14 pyroptotic DEGs with the sensitivity to many different drugs, and found that HMGB1, CASP3, CHMP7, and most drugs were negatively correlated, whereas IL-6, CHMP4C, CHMP4B, and large drug sensitivities were positively correlated (**Figures 10A, B**).

## Discussion

An increasing number of studies have shown that pyroptosis plays an important regulatory role in tumors; however, current research on the role of pyroptosis in bladder cancer remains unclear. This study screened 14 pyroptotic DEGs from cancerous and paracancerous tissues of BLCA. We found that imbalances in the expression of pyroptotic molecules might be related to the regulation of genomic variation. To develop new ideas and effective treatment targets for BLCA, it is necessary to formulate an efficient predictive model. At present, the pyroptosis risk scores have been developed to predict the prognosis and TME characteristics of cancer (2, 29). However, there is still a lack of research to systematically explore the correlation between pyroptosis-related features, TME features, and the molecular types of BLCA. This study developed and validated a novel pyroptosis risk score by using a combination of multiple independent BLCA datasets and the Xiangya cohort. The pyroptosis risk score can predict clinical outcomes, molecular subtypes, and TME characteristics and the therapeutic effect of chemotherapy, radiotherapy, ICB, and targeted therapy in BLCA.

Pyroptosis affects tumor proliferation, invasion, and metastasis (30). Some studies have found that high expression of GSDME in esophageal cancer causes cells to undergo pyroptosis (31). The pyroptosis risk score reflects the actual pyroptosis in the TME from different aspects. First, the pyroptosis risk score can be used to predict the prognostic and clinical features of BLCA. The higher the score, the worse the OS and the higher the tumor grade and stage. Second, we analyzed the differences in the enrichment scores of 50 landmark signaling pathways between the pyroptosis risk score groups and found that EPITHELIAL MESENCHYMAL TRANSITION and INFLAMMATORY RESPONSE were most abundant in the high-risk group, whereas PEROXISOME and ESTROGEN RESPONSE_EARLY were the most abundant signals in the low-risk groups. Chemotherapy is an important treatment option for metastatic bladder cancer (MBC). There is an urgent need to develop accurate chemotherapy predictors that can provide patients with precise treatment options. Our study found that the six most commonly used chemotherapeutic drugs in BLCA were the most sensitive in the high-risk group, indicating that patients with high-risk scores were more effectively treated with chemotherapeutic drugs.

Chen et al. found 28 genes related to cell death in BLCA (32). They found that patients in the high PyroScore group had better prognosis. Conversely, our study found that patients in the high-risk score group had worse prognosis. However, we consistently found that patients in the high scoring group were more sensitive to chemotherapy. However, our study and that of Chen et al. have several different focuses. First, the sets of pyroptotic genes selected were different between the two studies. Chen et al. summarized 28 genes related to pyroptosis in previous studies (33–35). In our study, we developed a pyroptosis risk score based on hallmark pyroptosis signatures with more robust enrichment analysis results than other published pyroptosis genomes. Second, Chen et al. A scoring system was constructed using the orthogonal rotation (PCA) method and named PyroScore (32). Therefore, we generated a pyroptosis risk score by integrating differential expression analysis, Cox analysis, and the LASSO algorithm. Third, Chen et al. did not analyze the relationship between the pyroptosis risk score and clinical outcomes, radiotherapy, or immune markers without systematic analysis. However, in our study, our system correlated the pyroptosis risk score with several TME immune signatures, such as TIICs, immune checkpoints, and TIS.

An increasing number of studies have focused on the tumor immune microenvironment (36, 37). Pyroptosis regulates the tumor immune microenvironment through various mechanisms. Pyroptosis regulates the expression of several immune-enhancing molecules, thereby forming the immune-promoting TME. For example, studies have found that GZM-B can also increase the number of macrophages, NK cells, and CD8+ T lymphocytes by cleaving GSDME, thereby activating antitumor immunity, activating caspase-3 in target cells, and inducing pyroptosis (38, 39). Two recent studies found that granzymes released by CD8+ T cells and NK cells can cleave GSDMB/E, thereby triggering tumor cell pyroptosis, and pyroptosis may be an important effector in anti-tumor immunity (38, 40). The study found that PD-L1 converts tumor necrosis factor α-induced apoptosis of cancer cells into pyroptosis, resulting in tumor necrosis (41). Studies have shown that pyroptotic cell death of cancer cells promotes dendritic cell activation and T cell infiltration and enhances anti-tumor immune responses by releasing high mobility group protein B1 (42). Further studies have shown that overexpression of GSDME results in enhanced drug sensitivity *in vivo* and *in vitro (* 43). All the above data are helpful for exploring the role of the pyroptosis risk score in predicting TME immune characteristics.

This study found that the pyroptosis risk score correlated with immune checkpoints (such as CD274, CTLA4, and PDCD1), TIS score, and anticancer immune cycle enrichment score (such as release of cancer cell antigens, T cell recruitment, CD8 T cell recruiting, Th1 cell recruiting, NK cell recruiting), and TIICs (such as CD8 T cells, NK cells, Th1 cells, and dendritic cells) were positively correlated, suggesting that there is higher anticancer immunity in the TME of patients in the high-risk score group. However, some tumor tissues have large numbers of immune cells (endothelial cells, mast cells, M2 macrophages, and quiescent T4 memory cells), which cannot penetrate the tumor and are forced to stay in the surrounding stroma. Anticancer immunity in the tumor microenvironment is considered an immunosuppressive state (44). This was because the pyroptosis risk score positively correlated with M2 macrophages (**Figure 5B**), which suppressed anticancer immunity, and the degree of infiltration was positively regulated by pyroptosis. It is well known that immune checkpoint inhibitors (ICIS) have achieved good results in tumor immunotherapy (10–12). Our study found that the pyroptosis risk score was positively correlated with the expression of many immune checkpoints such as CD274, CTLA4, and PDCD1. Therefore, anticancer immunotherapy such as ICB is more effective in patients with high-risk scores. In contrast, patients in the low-risk score group had significantly fewer TIS and immune checkpoints, which were negatively correlated, indicating that the TME had fewer immunotherapeutic targets. Therefore, the effect of ICB in patients in the low-risk score group was unsatisfactory.

This study had certain limitations. First, this study was conducted using a bioinformatics analysis. Although we have repeatedly validated these results in several public cohorts, we need to perform studies on the relevant mechanisms of pyroptosis *in vivo* or *in vitro*. Second, the clinical value of our pyroptosis risk score requires further validation through prospective clinical trials. Third, we did not determine the optimal cut-off value for the pyroptosis risk score.

In conclusion, we developed and validated a novel pyroptosis risk score that can effectively predict clinical outcomes and TME characteristics in BLCA. Pyroptosis risk score may contribute to the choice of BLCA treatment and enable patients to receive precise treatment. For patients in the high-risk score group, they may respond better to immunotherapy, chemotherapy, radiotherapy, and EGFR-targeted therapy. In contrast, patients in the low-risk score group may benefit from several targeted treatments, such as blockade of the PPARG, WNT-b-catenin, and FGFR3 networks.

## Data availability statement

The datasets presented in this study can be found in online repositories. The names of the repository/repositories and accession number(s) can be found in the article/**Supplementary Material**.

## Authors contributions

Conception and design: DD, FL, YW, ZO, and XZ. Provision of study materials or patients: ZW, YH, and CZ. Collection and assembly of data: YW, ZO, and ZL. Data analysis and interpretation: DD, YW, ZO, and FL. Manuscript writing: All authors. Final approval of manuscript: All authors.

## Funding

This work was supported by the grants from the Hunan Provincial Natural Scientific Foundation [2020JJ5916], the National Natural Science Foundation of China [82070785, 81873626, 81902592], and the Science and Technology fund project of Health and Family Planning Commission of Guizhou province (No.gzwjkj2022-100).

## Acknowledgments

We sincerely thank all participants in the study.

## Conflict of interest

The authors declare that the research was conducted in the absence of any commercial or financial relationships that could be construed as a potential conflict of interest.

## Publisher’s note

All claims expressed in this article are solely those of the authors and do not necessarily represent those of their affiliated organizations, or those of the publisher, the editors and the reviewers. Any product that may be evaluated in this article, or claim that may be made by its manufacturer, is not guaranteed or endorsed by the publisher.

## Supplementary material

Supplementary Figure 1 Expression patterns of these DEGs in the BLCA microenvironment from the single cell level.

Supplementary Figure 2 Expression patterns of these DEGs in the BLCA microenvironment from the single cell level.

Supplementary Figure 3 Expression patterns of these DEGs in the BLCA microenvironment from the single cell level.

Supplementary Figure 4 Expression patterns of these DEGs in the BLCA microenvironment from the single cell level.

Supplementary Figure 5 Expression patterns of these DEGs in the BLCA microenvironment from the single cell level.

Supplementary Figure 6 Expression variation of pyroptotic genes. **(A)** Fourteen pyroptosis genes had high mutation frequencies in UCEC, HNSC, STAD, COAD, and BLCA. Among them, the mutation frequency of the CASP8 gene was the highest, reaching 55%, particularly in UCEC. The darker the color, the higher is the mutation frequency, and vice versa. **(B)** Bubble chart shows the differential expression of these pyroptotic genes in the cancerous and paracancerous tissues of various tumors. Red indicates a positive correlation, and blue indicates a negative correlation. The darker the color, the higher is the correlation index. Bubble size indicates FDR. **(C)** Copy number variation pie chart distribution shows the types of copy number variation for these genes. Corresponding colors have annotations at the bottom, which indicate different types of copy number variation. **(D)** Bubble chart shows the correlation between the methylation of pyroptosis-related molecules and mRNA expression, with blue representing negative correlations and red representing positive correlations. Darker colors indicate a larger correlation index. The bubble size indicates the FDR. **(E)** Bubble chart showing the correlation between CNV and mRNA expression levels. Red indicates a positive correlation and blue indicates a negative correlation. The darker the color, the higher is the correlation index. The bubble size indicates the FDR.

Supplementary Figure 7 Correlation analysis of pyroptosis risk score with muscle invasive status and metastasis. **(A-C)** Relationship between the pyroptosis risk score and muscle invasive status in the TCGA-BLCA 、GSE32894 and Xiangya cohort. **(D-E)** Relationship between the pyroptosis risk score and metastasis in the TCGA-BLCA and Xiangya cohort. **(F)** Relationship between the pyroptosis risk score and histological type in the TCGA-BLCA.

Supplementary Table 1 Pyroptosis-related genes and detailed clinical information of four BLCA cohorts applied in our analysis.
