# Supplementary figures and images for: Robust pyroptosis risk score guides the treatment options and predicts the prognosis of bladder carcinoma
Source: Front Immunol. 2022 Aug 24;13:965469. doi: 10.3389/fimmu.2022.965469 (PMC9450692; doi:10.3389/fimmu.2022.965469)

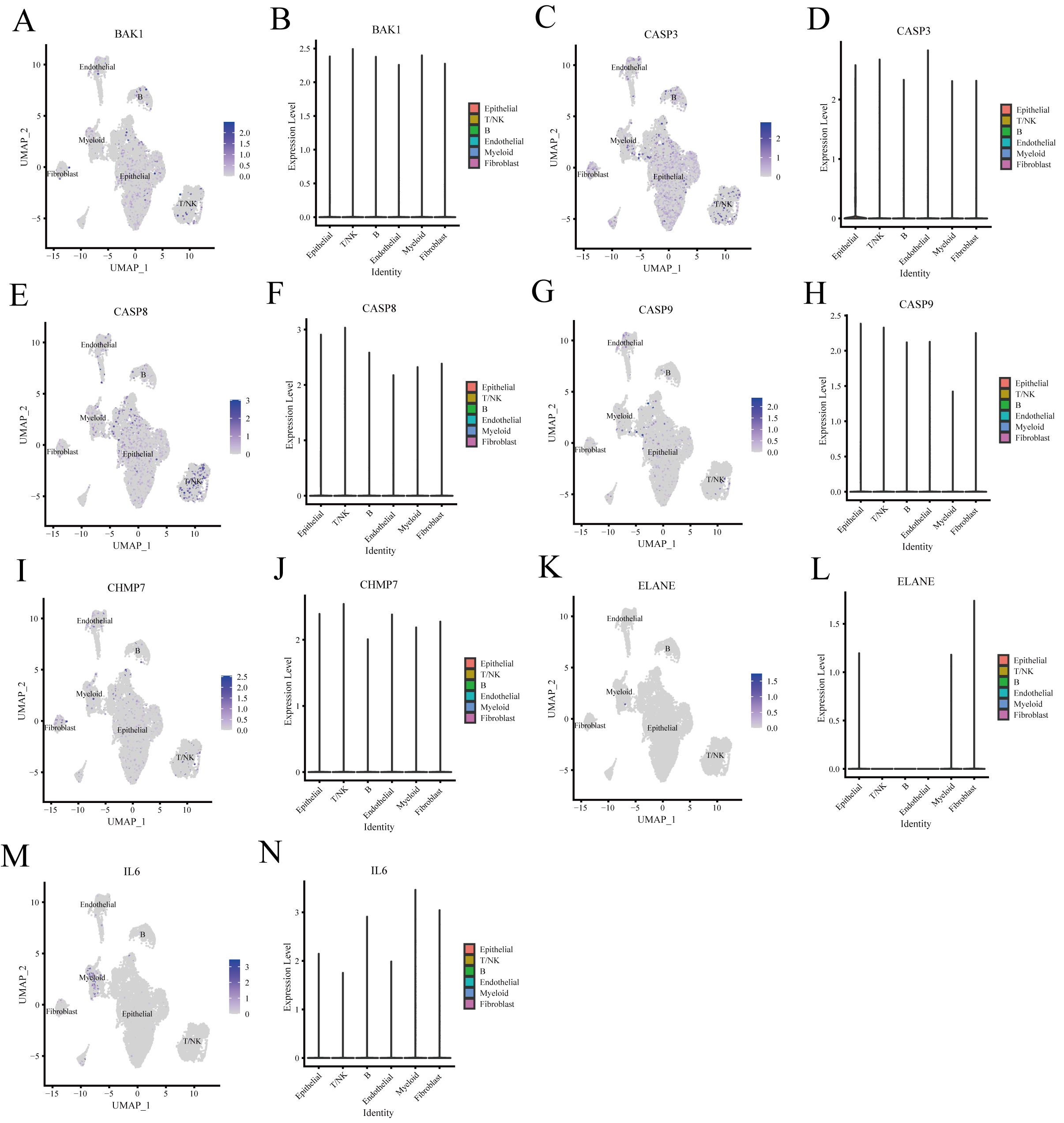

Supplement: Supplementary Figure 1 — Expression patterns of these DEGs in the BLCA microenvironment from the single cell level. [file Image_1.tif]

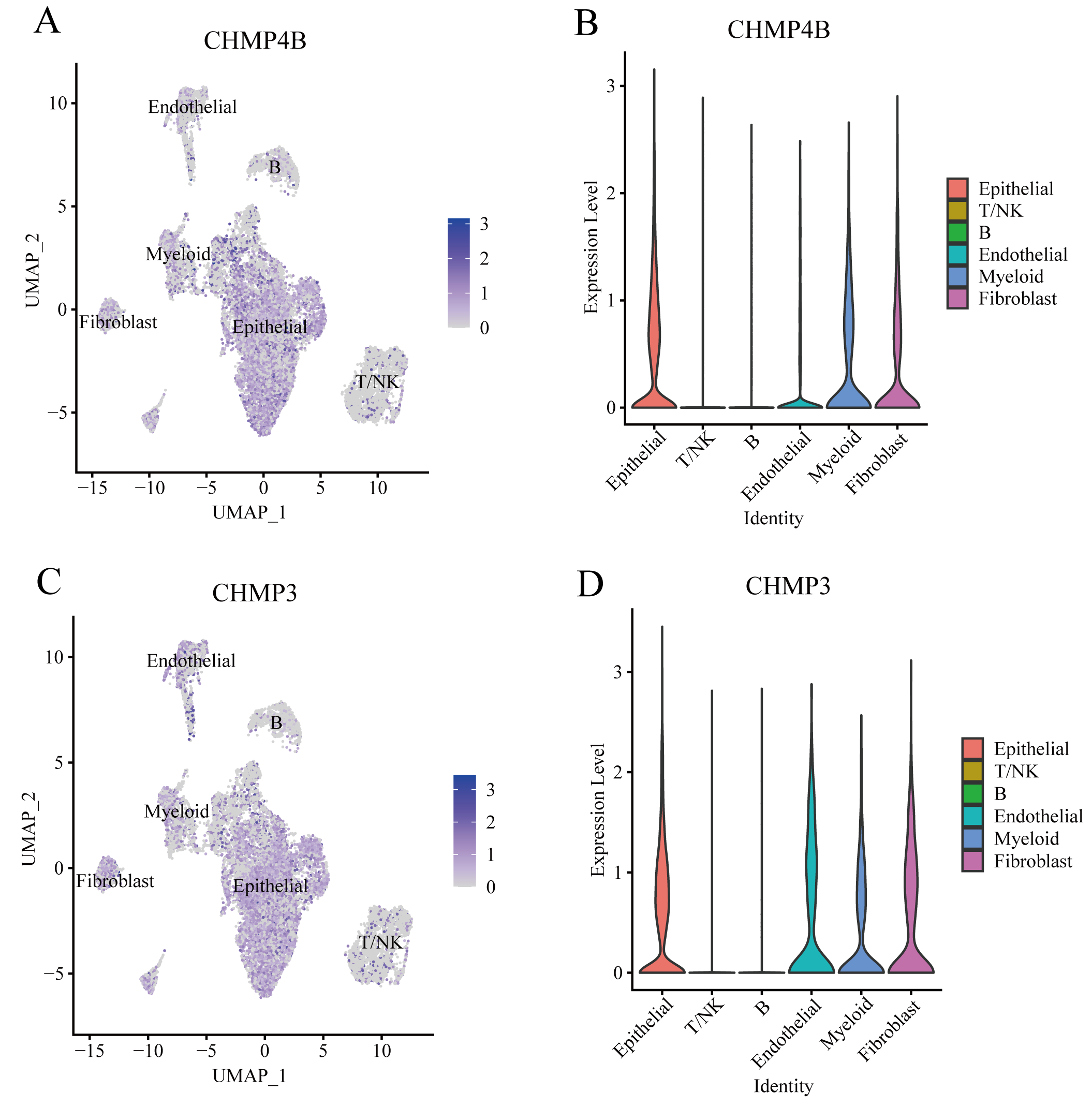

Supplement: Supplementary Figure 2 — Expression patterns of these DEGs in the BLCA microenvironment from the single cell level. [file Image_2.tif]

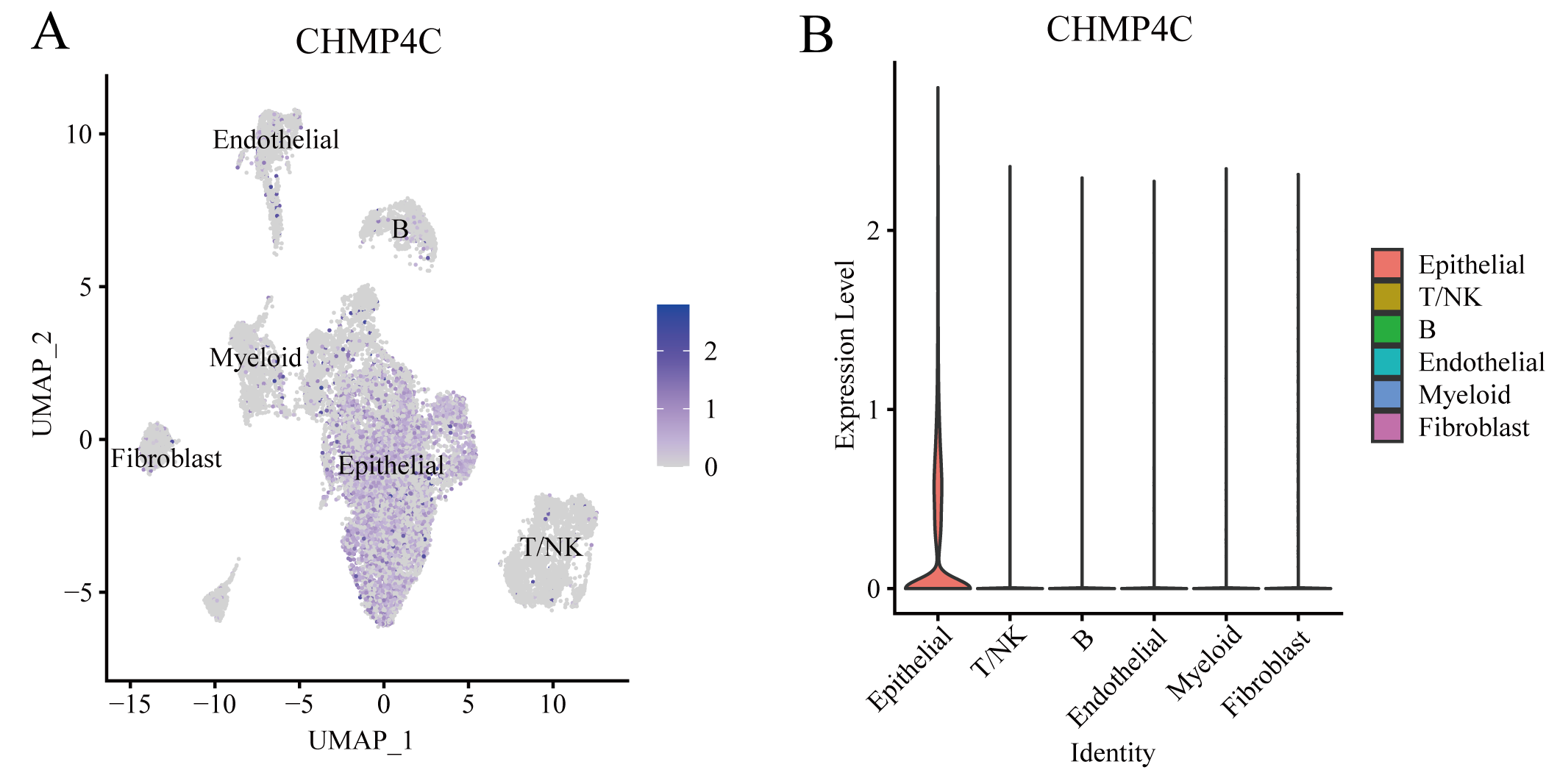

Supplement: Supplementary Figure 3 — Expression patterns of these DEGs in the BLCA microenvironment from the single cell level. [file Image_3.tif]

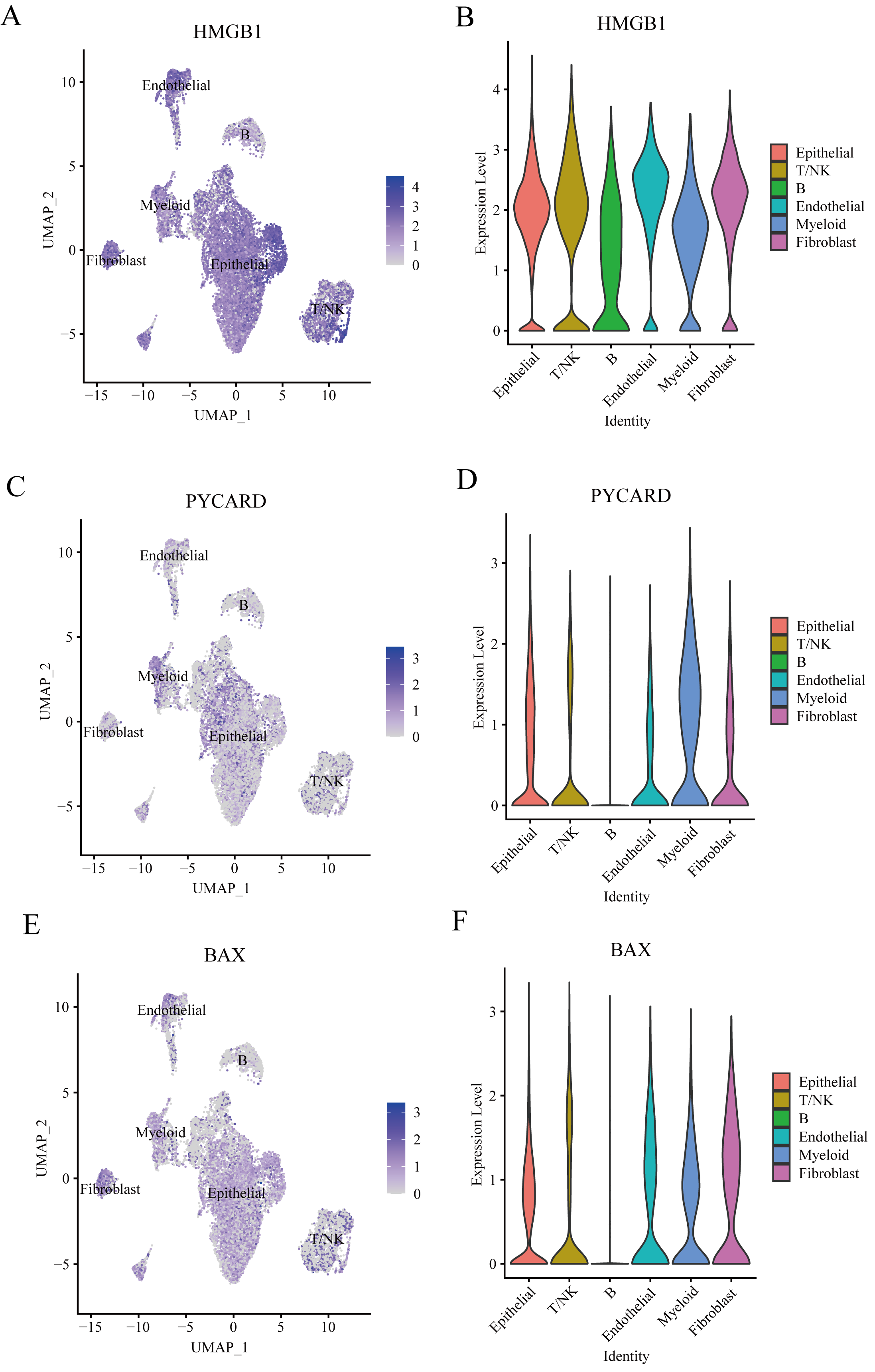

Supplement: Supplementary Figure 4 — Expression patterns of these DEGs in the BLCA microenvironment from the single cell level. [file Image_4.tif]

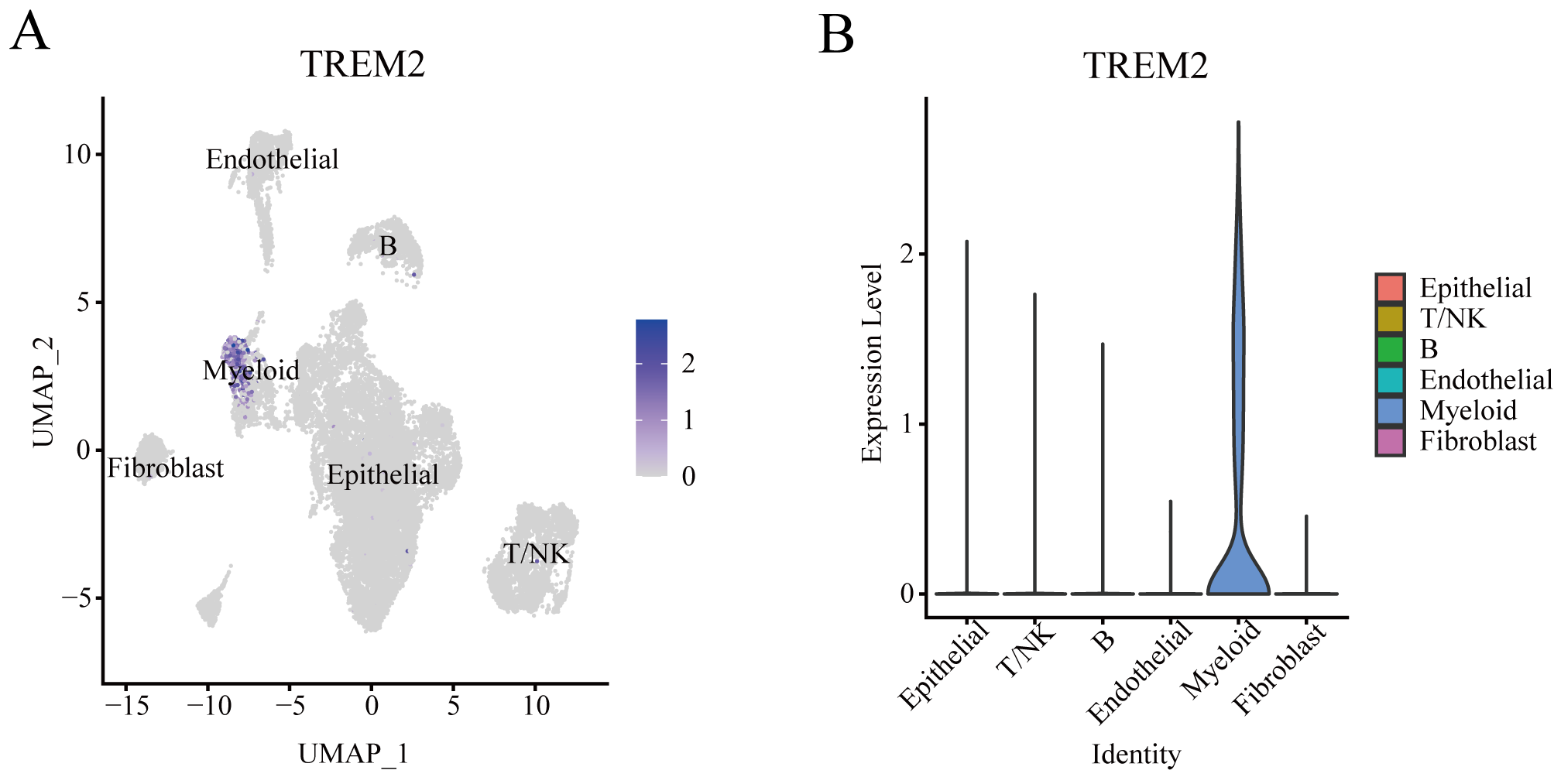

Supplement: Supplementary Figure 5 — Expression patterns of these DEGs in the BLCA microenvironment from the single cell level. [file Image_5.tif]

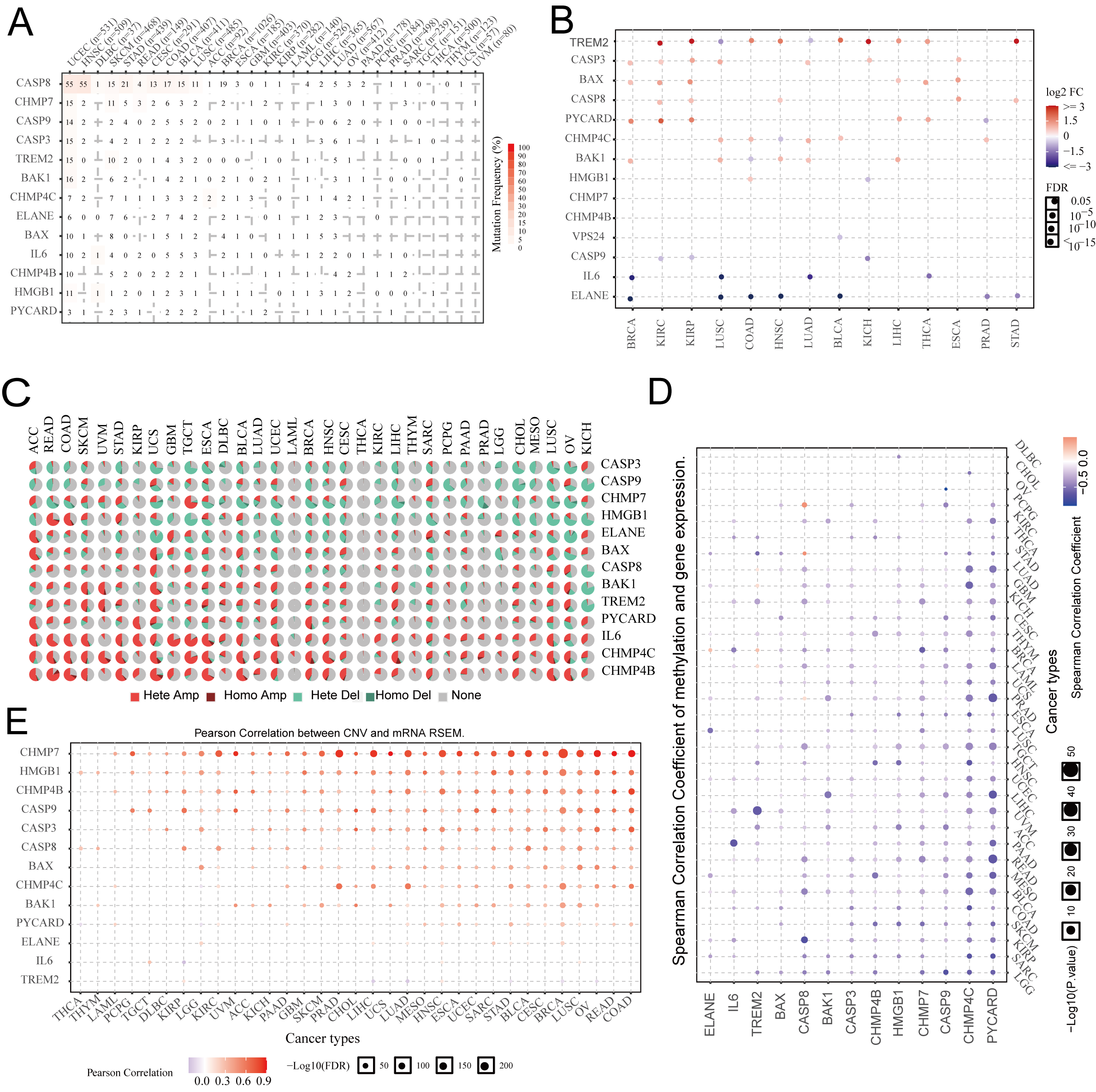

Supplement: Supplementary Figure 6 — Expression variation of pyroptotic genes. (A) Fourteen pyroptosis genes had high mutation frequencies in UCEC, HNSC, STAD, COAD, and BLCA. Among them, the mutation frequency of the CASP8 gene was the highest, reaching 55%, particularly in UCEC. The darker the color, the higher is the mutation frequency, and vice versa. (B) Bubble chart shows the differential expression of these pyroptotic genes in the cancerous and paracancerous tissues of various tumors. Red indicates a positive correlation, and blue indicates a negative correlation. The darker the color, the higher is the correlation index. Bubble size indicates FDR. (C) Copy number variation pie chart distribution shows the types of copy number variation for these genes. Corresponding colors have annotations at the bottom, which indicate different types of copy number variation. (D) Bubble chart shows the correlation between the methylation of pyroptosis-related molecules and mRNA expression, with blue representing negative correlations and red representing positive correlations. Darker colors indicate a larger correlation index. The bubble size indicates the FDR. (E) Bubble chart showing the correlation between CNV and mRNA expression levels. Red indicates a positive correlation and blue indicates a negative correlation. The darker the color, the higher is the correlation index. The bubble size indicates the FDR. [file Image_6.tif]

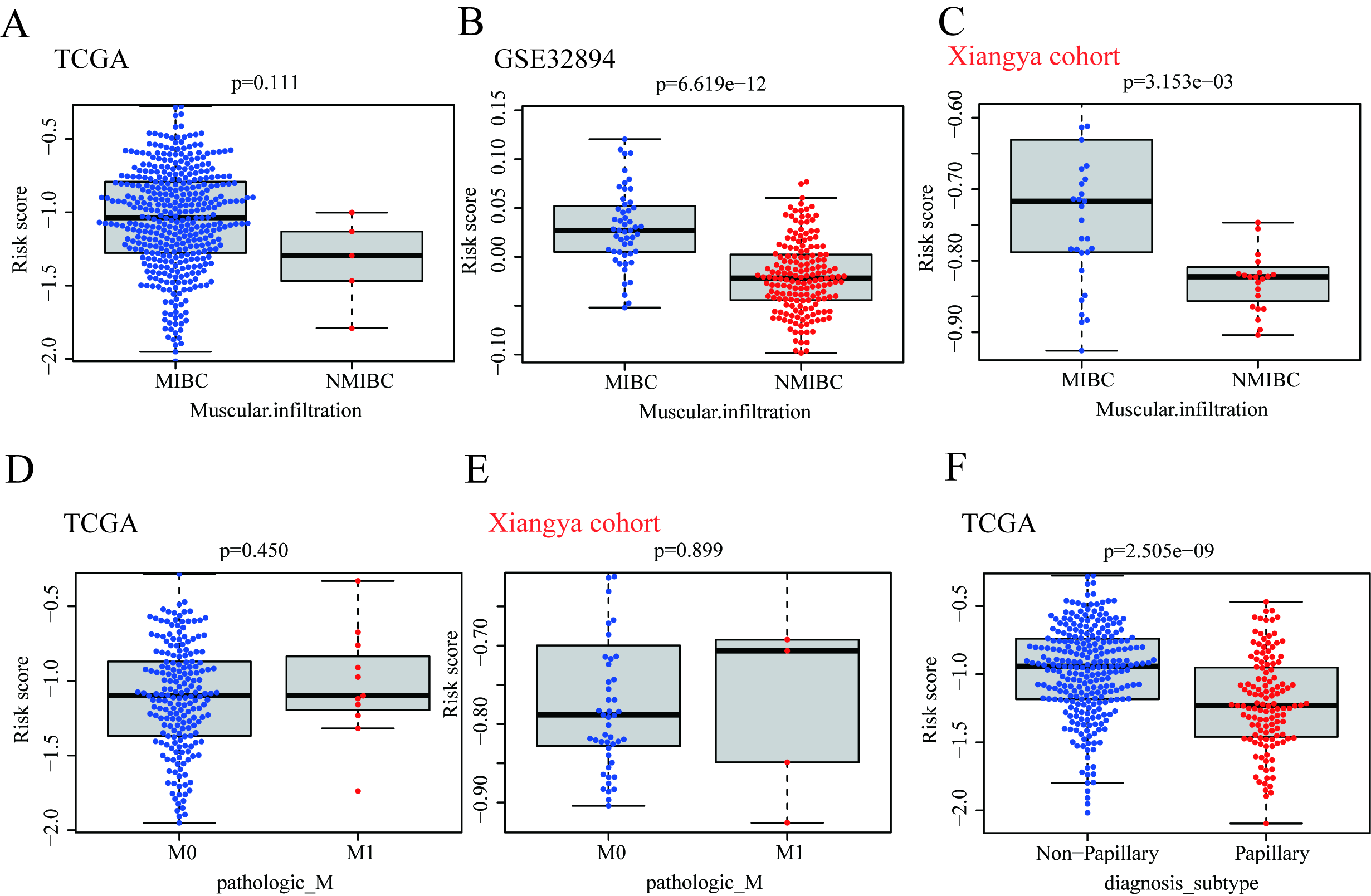

Supplement: Supplementary Figure 7 — Correlation analysis of pyroptosis risk score with muscle invasive status and metastasis. (A-C) Relationship between the pyroptosis risk score and muscle invasive status in the TCGA-BLCA 、GSE32894 and Xiangya cohort. (D-E) Relationship between the pyroptosis risk score and metastasis in the TCGA-BLCA and Xiangya cohort. (F) Relationship between the pyroptosis risk score and histological type in the TCGA-BLCA. [file Image_7.tif]
